# Developmental models of the carrion beetle *Thanatophilus rugosus* (Linnaeus, 1758) (Coleoptera: Silphidae)

**DOI:** 10.1038/s41598-021-98833-9

**Published:** 2021-09-29

**Authors:** S. Montoya-Molina, P. Jakubec, J. Qubaiová, M. Novák, H. Šuláková, J. Růžička

**Affiliations:** 1 Department of Ecology, Faculty of Environmental Sciences, Czech University of Life Sciences Prague, Kamýcká 129, 165 00 Praha-Suchdol, Czech Republic; 2 Police of the Czech Republic, Institute of Criminalistics Prague, P.O. Box 62/KUP, 170 89 Praha, Czech Republic

**Keywords:** Zoology, Entomology

## Abstract

Coleoptera are currently considered a fundamental tool to help solve criminal investigations, allowing forensic entomologists to estimate post-mortem intervals and obtain other ecology-related information. *Thanatophilus rugosus* (Linnaeus, 1758) is an important necrophagous beetle distributed through most of the Palaearctic region, where it is readily found on human bodies and animal carcasses. In this study, the new thermal summation models for all the developmental stages of *Thanatophilus rugosus* are provided. Beetles were reared at six different constant and ecologically relevant temperatures (12, 14, 16, 18, 20, and 22 °C), and their developmental times were measured. Thermal summation constants were calculated for each developmental stage (egg, three larval instars, post-feeding stage, and pupa).

## Introduction

Insect specimens found on a carrion during criminal investigations are a valuable piece of evidence when investigating time intervals relevant for the forensic sciences, e.g., Postmortem interval (PMI) and Pre-appearance interval (PAI)^1,2^. Using developmental information of Silphinae in medico-legal cases has nowadays proven to have many advantages when calculating the post-mortem interval^3^.

*Thanatophilus* Leach, 1815 (Silphidae: Silphinae) is distributed in Europe, Asia, North America, and Africa^4^. Forensic entomologists took interest in the genus only recently as they recognized its value for the field^5,6^. Many aspects of its ecology and morphology have already been studied such as the seasonal and daily rhythms^7,8^, preference to a particular soil type^9^, chemical ecology^10^, larval morphology and instar identification^11^. Recently, developmental models have been proposed for some of the *Thanatophilus* species e.g., *T. micans* (Fabricius, 1794)^5^, *T. capensis* (Wiedemann, 1821)^3^ and *T. sinuatus* (Fabricius, 1775)^12^. However, many more have yet to be studied, as this genus is one of the most diverse in the subfamily Silphinae with 24 described members to date^4^.

Along with other species colonizing carrion, *Thanatophilus rugosus* (Linneaus, 1758) is frequently found on carcasses during early spring in central Europe^13,14^ and throughout most of the year also in Southern Europe^15^. Despite the species being extensively collected on carcasses during criminal investigations across Europe, no developmental studies have been published so far.

In this article we present thermal summation models (TSM) based on the full developmental cycle of *T. rugosus*. Furthermore, we analyze whether the sex of an individual influences the developmental length, and whether the time proportions among certain instars are constant and independent. Additionally, survival rates among temperatures and developmental stages are evaluated.

## Results

### Development of *T. rugosus*

Overall, 713 individuals of *T. rugosus* were analyzed at six constant temperatures (12, 14, 16, 18, 20 and 22 °C). In all tested temperatures, individuals were able to complete the life cycle from egg to adulthood. In total, 526 out of 713 specimens reached adulthood.

The observed developmental lengths differed among temperatures. The duration of the development at the lowest temperature (12 °C) was 88.36 days (SD = 8.70; N = 88) while at the highest temperature (22 °C) was only 25.66 days (SD = 1.48; N = 70) (Table 2). The greatest proportion of the *T. rugosus* development cycle was spent in the Post–feeding (24%) and the pupal (33%) stages, while the shortest time was spent in the L1 and L2 stages (7–8%) in all studied temperatures.

### Survival rate and mortality

In total, the survival rate was above 70% in all the tested temperatures. Among all of the developmental stages, first larval instar (L1) and egg stage (Egg) had the highest in mortality rates, specifically 5% (N = 52) of deaths for L1 and 6.1% (N = 37) of deaths for Egg. The lowest mortality rate values were observed at the third larval instar (L3), post-feeding stage (PF) and pupal stage (Pupae), specifically 1.3% (N = 8) of deaths, 1.3% (N = 8) and 1.2% (N = 11) respectively. Second larval instar (L2) mortality rate was 3.8%. Survival rates values for each developmental stage are given in Table 1. Regarding the tested temperatures, the highest mortality rate was found at the highest temperature of 22 °C, where 25.5% of individuals did not complete their development. Increased mortality was found at 14 °C (19.2%) and 18 °C (19.5%) as well. Cannibalistic behavior in L3 larvae was observed towards the younger developmental stages of L2. Larvae cannibalized by the L3 were recognized due to the feeding marks on the ventral side, with their carcasses mostly comprising of the remnants of dorsal plates.

**Table 1:** Survival rates for all developmental stages of *T. rugosus* at six constant temperatures.

Developmental stage	Survival rate	Lower and upper CI (95%)
Egg	0.94 (37; 0.0098)	0.920–0.958
1st instar larva	0.89 (52; 0.0128)	0.864–0.914
2nd instar larva	0.85 (25; 0.0145)	0.823–0.880
3rd instar larva	0.84 (8; 0.0150)	0.809–0.868
Post-feeding	0.83 (13; 0.0155)	0.795–0.855
Pupae	0.81 (11; 0.0159)	0.782–0.845

The number of deaths followed by standard error shown in parentheses (*N; SE*).

### Thermal summation model

**Figure 1:**
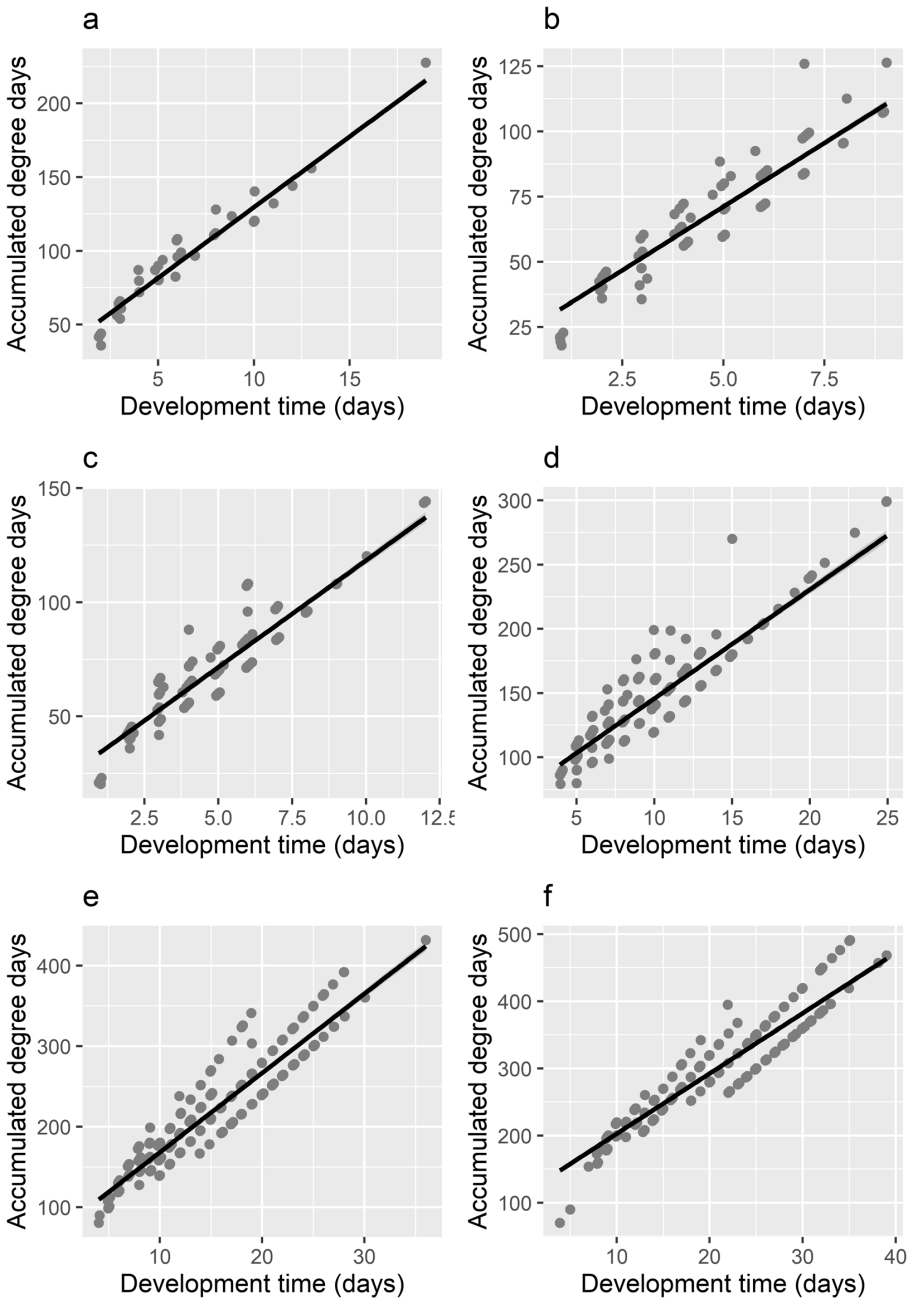
Ikemoto and Takai^30^ thermal summation model for all developmental stages of *T. rugosus*: (**a**) egg, (**b**) 1st larval instar, (**c**) 2nd larval instar, (**d**) 3rd larval instar, (**e**) post-feeding, (**f**) pupae. The points indicate the data used for the regression analysis.

Thermal summation models were established for all developmental stages (Fig. 1, Table 2). The accumulated degree days (ADD) and lower developmental threshold (LDT) values were calculated with expected errors (Table 3). To complete the whole development from the egg to the emergence of imago the individual needs to accumulate 362.75 ADD with a LDT set at 8.52 °C. Values coefficient of determination (R^2^), for all inspected models were above 0.85, indicating good fit on the data (Table 2).

**Table 2:** Mean developmental times of *T. rugosus* at six constant temperatures for each developmental stage (days, °C).

Temperature	Egg	1st instar larva	2nd instar larva	3rd instar larva	Post-feeding	Pupae	Total
12	11.60 (1.35; 105)	6.40 (1.11; 98)	6.82 (1.22; 98)	14.70 (3.10; 95)	20.80 (3.50; 92)	28.36 (3.58; 88)	88.03 (8.70; 88)
14	7.60 (1.07; 120)	5.30 (1.04; 105)	5.30 (0.86; 102)	10.48 (1.54; 99)	18.08 (4.65; 98)	25.17 (3.73; 97)	72.09 (8.02; 97)
16	5.81 (0.96; 107)	4.20 (0.70; 107)	4.16 (0.58; 106)	7.76 (1.14; 106)	11.52 (1.72; 105)	16.41 (1.96; 105)	49.85 (3.93; 105)
18	4.22 (1.12; 139)	3.17 (0.82; 115)	3.13 (0.78; 113)	7.14 (1.40; 112)	10.59 (2.40; 106)	13.65 (2.22; 101)	41.88 (4.82; 101)
20	3.40 (0.56; 14)	2.14 (0.38; 109)	2.40 (0.56; 100)	5.71 (0.95; 100)	6.70 (1.04; 99)	10.13 (1.02; 98)	30.47 (2.00; 98)
22	2.62 (0.63; 83)	1.81 (0.43; 82)	2.02 (0.65; 72)	4.75 (0.70; 71)	6.00 (0.83; 70)	8.60 (0.65; 70)	25.66 (1.48; 70)

Standard deviation followed by the number of observed specimens shown in parentheses (*SD; N*).

**Table 3:** Summary of the developmental constants for *T. rugosus* at six developmental stages.

Developmental stage	Temperature range of model (°C)	*R*2	*df*	*p*-value	ADD (°C)	LDT (°C)
Egg	12–22	0.942	663	> 0.001	34.191 (0.60)	9.532 (0.91)
1st instar larva	12–22	0.89	611	> 0.001	22.417 (0.60)	9.762 (0.13)
2nd instar larva	12–22	0.875	586	> 0.001	24.902 (0.64)	9.320 (0.14)
3rd instar larva	12–22	0.871	579	> 0.001	61.117 (1.25)	8.465 (0.13)
Post–feeding	12–22	0.91	566	> 0.001	70.080 (1.79)	9.820 (0.12)
Pupae	12–22	0.924	555	> 0.001	114.166 (2.03)	8.923 (0.10)
Complete development	12–22	0.944	555	> 0.001	362.758 (4.97)	8.528 (0.08)

Standard errors shown in parentheses.

### Effect of sex on developmental length

The effect of sex on the developmental length was calculated using a group of beetles that were able to complete development until the adult stage, from across all of the studied temperatures. The sex ratio (the number of males related to the number of females) was on average 1.067 (i.e. in favor of males); however, the probability of males and females in the sample did not differ significantly from equality (Exact binomial test, N = 253, 95% CI [0.44, 0.52], p value = 0.4842).

**Figure 2:**
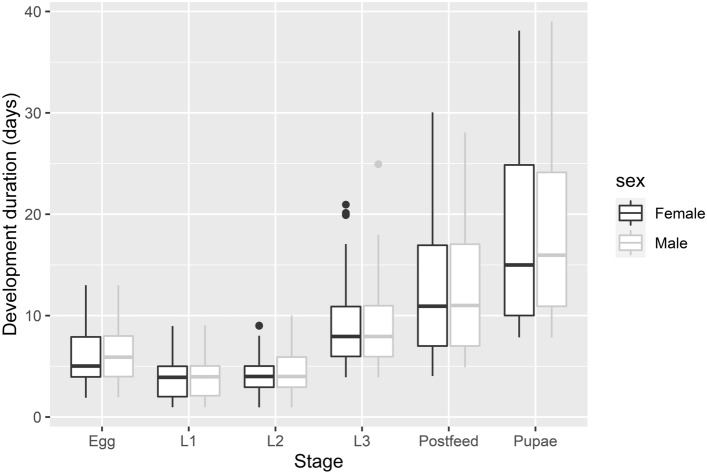
Developmental length differences between sexes. Horizontal lines within the boxes indicate median values; upper and lower boxes indicate the 75th and 25th percentiles, respectively. Whiskers indicate the values within the 1.5 interquartile ranges. Small dots are outliers. Females indicated in clear black boxes and Males in faded gray color boxes.

The developmental length was similar for both sexes throughout all of the developmental stages and even as a total (see Fig. 2). Comparison of the null and sex model by AIC values showed that the null model had a significantly lower AIC value (null AIC = 15,460.29, sex AIC = 15,464.73), therefore the information about the sex did not significantly improve the fit of the model.

### Developmental rate isomorphy—DRI

Dirichlet regression was applied to the individual development data for the Egg, L1, L2, L3, PF and the Pupae of *T. rugosus* reared under all studied temperatures. When comparing all three models (mod0, mod1 and mod2), the mod2 with quadratic effect of temperature had the lowest value of AIC (AIC = ** − **13,909.4), followed by the mod1 with temperature as the explanatory variable (AIC = ** − **13,839.0). On the opposite side was the mod0 implying DRI, which was accompanied with the highest value of AIC (AIC = ** − **13,638.2), suggesting the worst fit to the data. Therefore, we can conclude that the proportion of time spent at the individual developmental stages is not constant but has a quadratic effect with temperature.

## Discussion

This manuscript proposes the first thermal summation model for all the developmental stages of *T. rugosus*. This work complements the recent research on the Central European carrion beetles of forensic importance (see^11,12,16–19^). Moreover, the results provided are also applicable in countries outside Europe where *T. rugosus* occurs^20^.

Overall, the survival rate was above 70% (N = 526) in all the studied temperatures. These results suggest that the tested temperatures were not stressful or unfavorable. On the contrary, the survival rates indicate that all the tested temperatures were optimal for the beetle’s successful development, ensuring a more extensive data set for the TSM. We found that *T. rugosus* is likely to survive and perform slightly better at the lower intermediate temperatures (16–18 °C) rather than the high ones (> 20 °C). This could be associated to its natural seasonal activity in Europe^7,13,15^.

Developmental data at two constant temperatures were previously published by Ref.^21^, for species referred to as *Silpha rugosa* Linnaeus, 1758, which is an invalid taxonomic name for *T. rugosus*. However, *T. rugosus* has never been reported from Algeria where the study took place, nor northern Africa in general^20^, and its occurrence here is unlikely. We believe that the developmental information presented by Guerroudj and Berchi is actually referring to *T. ruficornis* (Kuster, 1851). *T. ruficornis* does occur in Algeria and can be misidentified as *T. rugosus,* due to the similar shape and texture of elytra^22^. Both species can be further separated morphologically, e.g., by the color of the antennomeres (combination of black and red in *T. ruficornis*, uniformly black in *T. rugosus*) and by the differences in shape of the posterior margin in the female’s abdominal tergum VIII (lateral emargination being deeper than the middle one in *T. ruficornis*, while equally emarginated in *T. rugosus*^22,23^). This means that the species was most likely misidentified and the developmental data in fact referred to *T. ruficornis* instead of *T. rugosus*. This would also explain the difference in our developmental data and those reported by^21^. They recorded that the development at 23 °C took 32 days, whereas our data suggests that the development at a lower temperature (22 °C) took on average 25.66 days, which represents a significant difference. This shows that correct identification of the insect species investigated in criminal sciences is critical for the precise calculation of the PMI because for even a closely related species, the physiological requirements may vary greatly.

The information provided by our TSM models and their parameters allow us to compare the thermal requirements of *T. rugosus* with other studied species from the genus and put them into perspective with their underlying ecology. It seems that even species that occur together do not share a similar lower developmental threshold. The LDT of *T. rugosus* (LDTT.rugosus = 8.528) differs considerably from its closest European sister species, *T. sinuatus* (LDTT.sinuatus = 9.85)^12^. However, total ADD are very similar for both species (ADDT.rugosus = 362.7 and ADDT.sinuatus = 360.4)^12^. When compared to the Afrotropical species, the thermal constants vary significantly. For *T. capensis*, the LDT and ADD values (9.04; 384.1)^3^ were relatively close to the ones of both *T. rugosus* and *T. sinuatus*^12^. Interestingly, *T. micans* represents a peculiar case, where its TSM values of LDT are the highest (LDTT.micans = 13.26) and ADD is the lowest (ADDT.micans = 197.97)^3^, when compared to the *Thanatophilus* species aforementioned (*T. rugosus, T. sinuatus* and *T. capensis*)^3,12^.

Honěk^24^ mentions that the species thermal constants show a negative relationship between LDT and ADD, therefore the species with the higher LDT requires a lower amount of ADD than the species with lower LDT. LDT and ADD values can be linked to environmental adaptations such as seasonality and temperature depending on the species distribution^24,25^. In practice, it is expected that the lower LDT values can be found in a species occurring in temperate regions such as *T. rugosus*, *T. sinuatus* and *T. capensis*. On the other hand, higher values of LDT can be expected for the species distributed throughout the tropical areas such as *T. micans*. The different values of LDT and ADD between *T. rugosus* and *T. sinautus* demonstrate their differences in seasonal activity, that regulate competition between these two closely related sympatric species^25^.

The LDT and ADD values also differ among other silphid species outside the genus *Thanatophilus*. In *Necrodes littoralis* (Linnaeus 1758) the LDT value (LDTN.littoralis = 8.49) is closer to the one of *T. rugosus*, however the ADD is much higher (ADDN.littoralis = 468.89), probably due to the larger size of the species. On contrary, in *Necrophila brunnicollis* (Kraatz 1877) both parameters vary significantly (LDTN.brunnicollis = 14.71) (ADDN.brunnicollis = 243.74) when compared to *T. rugosus*. In this case we could attribute the discrepancy to the adaptation to different climatic conditions as *N. brunnicollis* is a tropical species while *T. rugosus* is a temperate species.

Regarding the developmental times, differences were found between *T. rugosus* and other *Thanatophilus* species (Table 4). *T. rugosus* seems to develop faster at all temperatures compared to *T. sinuatus* as well as Afrotropical species *T. micans* and *T. capensis*. In addition, *T. rugosus* needs less time to develop also compared to other silphid species, like *Necrophila brunnicollis* (Kraatz 1877)^26^ and for *Necrodes littoralis* (Linnaeus 1758)^18^. In addition, our results confirm that the proportion of time spent in a particular developmental stage is dependent on the temperature. A faster development rate and the violation of the DRI could be suggested for species distributed in the temperate region as its developmental rate and growth can be adjusted based on the seasonal limitations and photoperiods^27^. Our findings are thus in accordance with the results already published in other forensically important species of Silphidae^12,26^.

**Table 4:** Comparison among the estimated developmental times of *T. rugosus* at three constant temperatures for five forensically important Silphid species.

	*T. rugosus*	*T. sinuatus *^12^	*T. micans *^3^	*T. capensis *^3^	*N. brunnicollis *^26^	*N. littoralis *^18^
14	72.09	78.23	110.68	77.11	–	85.09
18	41.88	43.44	44.90	46.14	74.09	49.30
22	25.66	29.67	28.16	32.92	33.43	34.70

Here we present the first thermal summation models for all developmental stages of *T. rugosus* reared at six constant temperatures (12, 14, 16, 18, 20 and 22 °C) under controlled laboratory conditions. The models allow *T. rugosus* to become another important tool for forensic entomology. The added benefit of including this species is its utility during the cold season when many other forensically important species are absent. Moreover, the data provided here can be used to validate PMI calculations based on other forensically relevant insects.

## Methods

### Laboratory colony establishment

Adult beetles of *T. rugosus* were collected using pitfall traps baited with pork muscle tissue (*Sus scrofa* Linnaeus, 1758) (Mammalia: Suidae) and beef muscle tissue (*Bos taurus* Linnaeus, 1758) (Mammalia: Bovidae). The sampling was conducted in the Czech Republic in 2020. Specimens were sampled at three localities: Stará Lysá (50° 13′ 08.0′′ N, 14° 48′ 25.5′′ E), Slapy (49° 47′ 31.9′′ N, 14° 23′ 52.7′′ E) and Prague—Lysolaje (50° 07′ 33.0′′ N 14° 21′ 44.0′′ E). Due to the seasonal and ecological preferences of the species, individuals were collected between April and May by placing the traps in open areas, in habitats such as harvested agricultural fields or grasslands^15,28^.

The methodology used for larval breeding in this study follows Montoya-Molina et al.^12^ and was originally based on Ridgeway et al.^3^, with modifications described in detail there. The larvae of the first and second instar from the same clutch of eggs were kept together under the condition that they were at the same developmental stage. If some larvae molted before the rest of the group, they were moved to separate Petri dish. Developmental milestones (egg, 1st instar larva, 2nd instar larva, 3rd instar larva, Post-feeding stage) and pupae) were identified based on morphological features described by Novák et al.^16^. The whole breeding process was monitored daily.

Colonies were kept inside climatic chambers (custom made by CIRIS s.r.o.) at six constant temperatures (12, 14, 16, 18, 20 and 22 °C) and 16 h of light and 8 h of dark photoperiod regime, maintained by a fluorescent light (Osram L 8W/640) to simulate the light conditions during the species breeding season. The temperatures used were based on previous rearing of this beetle by Jakubec et al.^11^.

### Mortality rate analysis

Survival data were evaluated by a nonparametrical log rank test using the function survdiff from the R package survival^29^. The effect of temperature on mortality was investigated at all experimental temperatures (12, 14, 16, 18, 20 and 22 °C). We measured the mortality of immature individuals starting with the egg and the data were right hand censored at the moment of adult beetle emergence. Bonferonni correction was used for post–hoc comparisons of the treatments.

### Thermal summation model

To model the relationships between the developmental time and the temperature for all the developmental stages, a linear regression model defined by Ikemoto and Takai^30^ was applied. Both estimated parameters of the TSM (LDT and ADD) are defined as a slope and intercept of the linear regression respectively. Therefore, both LDT and ADD can be easily calculated along with their respective standard errors^30^.

### Effect of sex on development length

Across all temperatures, only the individuals that have completed the development until the adult stage were selected for the study, as only these specimens could be sexed.

To assess the potential differences in developmental time between males and females, two linear mixed effect regression models were fitted (null and “sex” model). Response variables of both models were the developmental lengths of the six stages (Egg, L1, L2, L3, PF and Pupae) and their total. Null and the alternative model also shared two fixed effect explanatory variables: temperature (12, 14, 16, 18, 20 and 22 °C) and developmental stage. Because the measurements of the development times were done repeatedly on the same individual, the identity of that specimen had to be incorporated into the analysis, therefore we used it as a random effect in both models. The only difference between the alternative and the null models was fitting the latter with sex as a fixed effect explanatory variable. The fit of these two models was compared via AIC criteria to find out if the information about sex does improve it significantly.

### Developmental rate isomorphy (DRI) and sex ratio

The presence of developmental rate isomorphy (DRI) was tested using Dirichlet regression as suggested by Boukal et al.^27^. The isomorphy hypothesis suggests that the development rates do not depend on the temperature. For the analysis, a proposed null model (mod0) was tested without the explanatory variables. The mod0 implies that the proportions of time spent in individual instars are constant and independent of other factors (true presence of the DRI in the species). Two alternative models were fitted, one with temperature as the explanatory variable (mod1) and the other with expected quadratic effect of temperature (mod2). Both alternative models suggest that the DRI is not present in the species. Relative quality of each model was evaluated by Akaike information criteria (AIC), to allow comparison. The one with the lowest value was considered as the most appropriate description of the underlying relationship.

A Binomial test was conducted to determine whether the observed sex ratio for the species is significantly different from the expected one (1:1).

### Data management and analysis

All data management and analyses were carried out using the R statistical program^31^. Additionally, we used a “lme4” package for fitting mixed effect models and visual outputs were processed via packages “ggplot2” and “sjPlot”^32–34^.
